# Supplementary material for: Comparison on Major Gene Mutations Related to Rifampicin and Isoniazid Resistance between Beijing and Non-Beijing Strains of Mycobacterium tuberculosis: A Systematic Review and Bayesian Meta-Analysis
Source: Genes (Basel). 2022 Oct 13;13(10):1849. doi: 10.3390/genes13101849 (PMC9601453; doi:10.3390/genes13101849)
Supplement: Supplementary file 1 [file genes-13-01849-s001.zip › Supplementary file S1__Search engines & terms.pdf]

Search engines: Google Scholar, PubMed, ResearchGate, ResearchGate, Cochrane Library and Chinese National Knowledge Infrastructure (CNKI) Database.

Search terms: MTB AND Beijing AND non-Beijing AND gene mutation AND MDR, or RIF, or INH drug resistance; *rpoB* mutation AND Beijing AND non-Beijing AND MDR, or RIF; *katG* mutation AND Beijing AND non-Beijing AND MDR, or INH; *inhA* mutation AND Beijing AND non-Beijing AND MDR, or INH; *oxyR-ahpC* mutation AND Beijing AND non-Beijing AND MDR, or INH.
